# Vascular surgery trainee retention in the UK: how many leave and why? A survey of trainee and Training Programme Directors’ perceptions

**DOI:** 10.1186/s12909-021-02668-x

**Published:** 2021-04-26

**Authors:** Eleanor Atkins, Sandhir Kandola, Frances Kent, Olivia McBride, on behalf of the Rouleaux Club, the UK vascular surgery trainee association

**Affiliations:** 1 Norfolk and Norwich University Hospitals NHS Trust, Norwich, UK; 2 Royal Liverpool and Broadgreen Hospitals NHS Trust, Liverpool, UK; 3 Royal Infirmary of Edinburgh, Edinburgh, Scotland, UK

**Keywords:** Vascular surgery, Surgical training, Trainee retention

## Abstract

**Background:**

It is currently not known how many trainees leave vascular surgery, and their reasons for doing so are unclear. This paper is the first to publish the number of UK trainees leaving the training programme and interrogates their reasons for doing so.

**Methods:**

An email survey was distributed to current and recent Training Programme Directors (TPDs) to quantify the number of trainees resigning between 2013 and 2019. Trainees resigning a National Training Number (NTN) were surveyed regarding their reasons for doing so.

**Results:**

Since 2013, 23 UK vascular surgery trainees have resigned NTNs, representing 15.4% of the 149 NTNs awarded between 2013 and our analysis. Reasons for leaving, as relayed by TPDs, included availability of an academic career, geography, health and many other reasons classified as “work-life balance” factors. Data from the trainees surveyed also highlighted work-life balance but also identified pressures within the training system and NHS.

**Conclusions:**

UK data of this sort has not previously been available. The authors’ primary recommendation is that prospective data collection on trainee retention is carried out, with structured exit interviews with trainees who decide to leave. Our secondary recommendations include improvements to the inter-deanery transfer process and early realistic exposure to vascular surgery for junior doctors to improve trainee retention rates in vascular surgery.

**Supplementary Information:**

The online version contains supplementary material available at 10.1186/s12909-021-02668-x.

## Background

The ageing population is growing, and the prevalence of conditions such as diabetes, peripheral arterial disease and other co-morbidities requiring treatment by vascular surgeons is increasing, predicting a rise in demand for vascular surgeons by 67% by 2029. Due to this, The Vascular Society of Great Britain and Ireland (VSGBI) Workforce Survey in 2018 indicated that 138 new vascular surgeons and 198 new trainees will be needed across the UK to achieve and sustain the VSGBI recommended ratio of 1 vascular surgeon per 100,000 population [1].

In addition to the increased demand, consultants are also leaving the specialty. Among vascular surgery consultants responding to the 2018 Workforce Survey, 34% stated that they were “extremely likely” to retire in the next 10 years. More recently, the Royal College of Surgeons survey administered after the changes to NHS pension tax rules indicated 68% of consultant surgeons were considering early retirement, and 69% had already reduced their working hours [2]. Retirements and reductions in working hours will impact the number of vascular surgeons serving the UK population.

The increased demand for vascular services, combined with consultant retirements and reductions in working hours necessitates an increase in the number of doctors training to become vascular surgeons. In the UK, the vascular surgery training programme has recruited 19 to 33 trainees per year since its inception in 2013 (prior to this vascular surgery was a subspecialty of general surgery). However, not all doctors awarded a National Training Number (NTN) in vascular surgery go on to complete training. There is therefore a pressing need to identify how many trainees do not complete training, and their reasons for doing so, to encourage future retention of vascular trainees and consequently maintain the number of future consultants.

Information about trainee retention rates in the UK is not centrally recorded or published. In addition, data specific to vascular surgery would not have been available prior to 2013, when vascular surgery remained a subspecialty of general surgery. In 2016, Hampton et al. asked UK deaneries to provide data on leavers from higher surgical training. They received subspecialty-specific responses from 6/13 deaneries, and found the rate of trainee attrition for general surgery during 5 years’of surgical training to be 1.09% [3]. In the US Chen et al. showed an 0.4–2.5% attrition rate per year from vascular surgery training, and showed that specific training programmes in vascular surgery had greater trainee retention than more generalised programmes [4]. A comprehensive study of all US general surgery residents showed that 3% resigned per year, leading to a cumulative loss of 19.5% of their residents during the training programme [5].

In this paper, we are the first to quantify the retention of UK higher surgical trainees in vascular surgery since the inception of the new training programme in 2013. This also represents the first complete nationwide data from a single surgical subspecialty. We qualitatively assessed the reasons for trainees leaving training programmes using surveys of both trainees and Training Programme Directors (TPDs) and hope this new information will drive improvements to recruitment and retention to surgical training programmes.

## Methods

A Freedom of Information (FOI) request was submitted to Health Education England (HEE) asking how many trainees had resigned a NTN in vascular surgery since 1st August 2013, as well as in general surgery, trauma and orthopaedic surgery, ENT, plastic surgery, general medicine, cardiology, gastrointestinal medicine, respiratory medicine, healthcare of the elderly and rheumatology for comparison. The chair of the vascular Specialist Advisory Committee (SAC) was contacted by email to ask how many trainees had resigned an NTN in vascular surgery since 1st August 2013.

An email survey written for this project was distributed to the TPDs of all 13 UK deaneries (Yorkshire and the Humber, West Midlands, Wales, Wessex/Thames Valley, South West, Scotland, Northern Ireland, North West, North East, London, Kent/Surrey/Sussex, East of England and East Midlands). If the TPD was only recently in post, the survey was sent to a recent predecessor in the role in addition to the current TPD. The survey comprised 8 open and closed questions, some with immediate follow up questions. (Additional file 1). Guidance was given on maintaining confidentiality by not providing identifiable trainee details.

Although it is likely that TPDs would have access to contact details of trainees resigning NTNs, passing on their details would have contravened data protection legislation. In addition, we were keen for our enquiry to come from someone independent of the training infrastructure to encourage trainees who resigned to feel they could communicate their ideas openly and candidly. Trainees who resigned a vascular NTN awarded between 2013 and 2019 were identified by Rouleaux Club (the national association for vascular trainees) committee members and their contacts, and news of the survey communicated to them through an initial word-of-mouth enquiry from a trainee they knew. Once the former trainee verbally consented to participate in the study they received an email questionnaire written specifically for this project (Additional file 2) which they completed and returned by email. The questionnaire comprised several open questions, to allow for former trainees to express their own ideas and to maximise the breadth of information gained, given the anticipated small number of responses.

Former trainees consented to participation by email and were informed their responses would be anonymised. Former trainees’ names, ages and geographical locations were not recorded due to small number of participants and a single author (SK) analysed and stored results to preserve anonymity. Analysis was performed using an Excel database. Broad themes and specific issues were identified from each former trainee and TPD questionnaire using open coding techniques.

## Results

### Freedom of information and enquiries to SAC

The Freedom of Information request to Health Education England was unable to be granted. HEE confirmed that while they do hold the data on trainees leaving medical and surgical specialties, it is not in a centrally retrievable or easily accessible format and collating it would exceed the 18 working hours deemed appropriate for FOI. The enquiry to the vascular SAC revealed that they hold no information about the numbers of trainees resigning training numbers.

### TPD survey

The data collected from Training Programme Directors (TPDs) survey responses indicated that 23 trainees have resigned National Training Numbers (NTNs) since 1st August 2013. This represents 15.4% of vascular surgery NTNs awarded since 2013 (number awarded 149). Different TPDs maintained different levels of confidentiality, and in order to maintain this we present the data nationally.

Of 20 trainees for whom curriculum information was given, 15 were on the new curriculum. Of 20 trainees for whom gender information was given, equal numbers were male and female. Of 16 trainees for whom ST level information was given, nine left in ST3 or ST4, and seven left in ST5, ST6 or ST7 (Table 1).

**Table 1:** Trainees leaving vascular surgery training. Overall, 15.4% of vascular surgery trainees resigned an NTN between 2013 and 2019. Most of these were appointed since 2013, and were therefore on the new vascular curriculum (separate from general surgery training). More trainees resigned during the first 2 years of training compared to the final 4 years

Total trainees leaving 2013–2019	23/149 (15.4%)
Trainees on new vascular curriculum (appointed since 2013)	15/20 (75%)
Grade when resigned NTN	
ST3–4	9/16 (56%)
ST5–8	7/16 (44%)

**Fig. 1:**
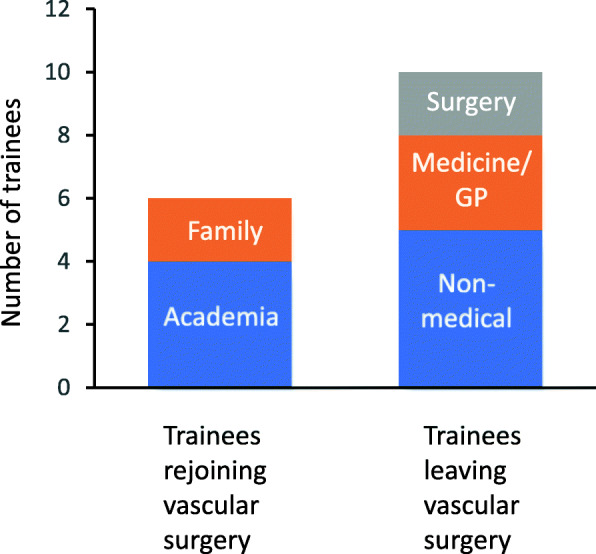
Trainees resigning NTNs rejoined vascular surgery training or sought alternative careers. Of the 6 trainees rejoining vascular surgery training, 4 did so for academic reasons (academic training not being available in their original region) and 2 needed to change the geographical location of their training for family or social reasons. Of the 10 leaving the specialty, 5 left a career in medicine altogether, 3 chose specialties outside of surgery and 2 changed to a different surgical subspecialty

Data on onwards jobs were given for 16 trainees. Six stayed in vascular surgery; four to take up academic posts and two re-applied via the ST3 application process for a new NTN in a different region. Two left to go to another surgical specialty, three to go to a medical specialty or general practice, and five left medicine altogether (Fig. 1).

Reasons for leaving, as identified by the TPDs, can be seen in Table 2. No training issues were identified that led to resignation, and no bullying or harassment was identified by the TPDs.

**Table 2:** Factors identified by TPDs and trainees contributing to the decision to leave higher specialist vascular surgery training. TPDs identified work-life balance and geographical factors as factors influencing trainees’ decision to leave training. Issues related to system pressures within the NHS or UK training system, such as high workload and demands of service provision over training, were not mentioned by TPDs, suggesting a lack of awareness of the contribution of these factors

	TPDs	Trainees
**Geography**		
Availability of an academic career	✓	✓
Family/social reasons to change region	✓	✓
**Work-life balance**		
Unpredictable hours	✗	✓
Fatigue	✓	✓
Time spent with family	✓	✓
Non-clinical work (research/governance)	✓	✓
Long commutes	✗	✓
**System pressures**		
Managing multiple tasks	✗	✓
Lack of senior/junior support	✗	✓
Service provision prioritized over training	✗	✓
Lack of satisfied/fulfilled role models	✗	✓
**Money**	✓	✗
**Health**	✓	✗

Trainees’ reasons for leaving were discussed with their TPDs, but formal exit interviews did not take place in all but two deaneries. Time spent Out Of Programme (OOP) for experience, research or as a career break was used in several deaneries to allow trainees space and time to make a decision on continuing in training. Less Than Full Time training (LTFT) was not mentioned as part of the discussions held by TPDs.

### Survey of trainees who resigned an NTN

In all, 12 trainees who resigned a 2013 vascular surgery curriculum NTN were contacted and 9 provided responses to the survey questions, amounting to 39% of all trainees resigning an NTN since 2013. All 9 trainees resigned at ST3–5 and all had a minimum of 6 months’ exposure to vascular surgery at SHO level (median 10 months, range 6–20 months at FY2, ‘F3’ or core surgical training). Of the 9 survey responders, 4 trainees had reapplied and secured a different vascular NTN and 5 had left vascular surgery for an alternative career.

Of those trainees who reapplied for vascular training and secured a new NTN, the reason for this was entirely geographical. These trainees needed to change the location of their training, in 3/4 cases for family/personal reasons and in 2/4 cases to pursue academic training (which is not provided in all deaneries). All these 4 trainees attempted to arrange an Inter-Deanery Transfer (IDT) but were unable to do so. All trainees who reapplied for vascular surgery NTNs in a different geographical location suggested that the IDT process be made simpler and more transparent.

Of the 5 trainees leaving the specialty entirely, 4 cited reasons related to work-life balance. A compilation of the reasons these trainees mentioned included unpredictable hours, fatigue and lack of rest post on-call, long commutes and a need to continue to work when at home. Whichever reason(s) the trainee suggested, a negative impact on the quantity of time they spent not working and their enjoyment of life outside of work was percieved. These trainees found that the example set by their senior colleagues and trainers suggested to them that this would not improve as their career progressed. Work-life balance was also recognised by TPDs as a factor contributing to trainee resignations in their survey responses. Trainees suggested that more flexible assignment of rotations and working patterns, including increased use of less-than-full-time training might be a helpful way to address this, although only one of the trainees thought this might have had an impact on their decision to leave the specialty.

Of the 5 trainees leaving vascular surgery, 3 cited reasons related to system pressures. These included too high a clinical workload, insufficient junior and senior doctor support, prioritisation of service provision over training, a lack of support and recognition by trainers and senior doctors appearing dissatisfied or unfulfilled. This factor driving trainees to leave training programmes was not cited in the TPD survey response, suggesting TPDs were unaware of these issues’ contribution to a trainee’s decision to leave vascular surgery. Trainees suggested that gaining a more realistic impression of the working patterns of registrars and consultants in vascular surgery prior to committing to the specialty might have helped them to select a different specialty prior to starting higher surgical training in vascular surgery.

## Discussion

Our data show the first complete trainee attrition rate for a surgical specialty in the UK, and the first for vascular surgery. Over 5 years, 15.4% of vascular surgery NTNs appointed between 2013 and 2019 resigned. While this seems significantly higher than the previously published data for surgical trainees in the UK [3], data from the US and Australia are similar [6–8] in spite of significant differences between healthcare systems. More UK vascular surgery trainees resigned NTNs at an early stage of training (ST3 or 4), which is consistent with US data, which reports more trainees left US general surgical training during internship [5, 7].

Our 15.4% attrition rate is, however, artificially inflated by the several trainees re-joining vascular surgery training after resigning their NTN. Six trainees in total remained in the specialty, having re-applied through the ST3 application process to gain an NTN in a different deanery or to include academic training, taking on risk to their career in the process. The trainees’ survey responses clearly stated the current IDT system is not working for vascular surgery trainees, and current training programmes are too inflexible to accommodate aspiring academic vascular surgeons. We recommend, therefore, that the difficulties in the IDT system be investigated and the process improved for trainees, enabling them to train in a desired region or academic discipline without having to resign an NTN and reapply, a process fraught with risk to job security and career progression.

Of the gender data for vascular trainees leaving the specialty, equal numbers of male and female leavers were identified. However females only represent 30% of vascular trainees overall [9], making females over-represented among those who left the specialty. This is in agreement with some US and Australian data, which showed a higher proportion of female trainees leaving surgical specialties [6, 8]. As the proportion of female trainees entering training increases (female core surgical trainees in all surgical specialties reached 40% in 2018 [9]), it is concerning that if female trainees are more likely to resign, as this may mean in future more surgical trainees resign NTNs . We have not explored whether the factors driving trainees to resign NTNs affect female trainees disproportionately; this clearly merits investigation in future.

A study of US surgical residents considering leaving training cited poor sleep, long hours and an undesirable future lifestyle as factors [10], and these work-life balance or lifestyle related issues were cited by trainees who eventually left [11]. Whilst working conditions and hours are markedly different between Europe and the US, a study from the Netherlands of trainee attrition across hospital specialties also found that work-life balance, job content and workload to be the main reasons trainees leave training programmes [12]. Again, in Australia and New Zealand poor lifestyle and quality of life were also the main reasons trainees considered discontinuing surgical training [13]. It seems that surgery across the world places demands on trainees’ lives outside of work and causes some to reconsider surgery as a career.

Both trainees and TPDs identified work-life balance issues as contributors to the decision to leave vascular surgery. Trainees who left surgical training programmes believed increasing opportunities for LTFT training might also help retain trainees due to improvements in work-life balance, however TPDs did not discuss LTFT training with the trainees who left. A higher proportion of LTFT trainees are female, so increasing access to this may improve retention of female trainees.

In the US, dissatisfaction with operative experience contributed to trainees leaving surgery [11] and in the Netherlands, the job content and workload were factors in trainees’ decision to leave [12]. These issues are analogous to the system pressures we identified, suggesting that these are not unique to the NHS or UK training system. Conversely, data from the US suggest financial factors contribute to the loss of surgical trainees [14], something not highlighted by either trainees or TPDs in our work. We identified that issues affecting trainees related to system pressures were not known to TPDs. We suggest that conducting formalised exit interviews with trainees deciding to resign would help increase TPDs’ awareness of issues involved locally. Nationally, implementation of the recent GMC-led recommendations within the ‘Caring for Doctors, Caring for Patients’ document [15] attempts to improve the working lives of doctors in training, which may positively impact trainee retention.

Trainees themselves suggested that having a realistic expectation of the workload and lifestyle of surgeons might have helped them to make a more appropriate career decision at an earlier stage, and work from the US suggests that if this is the case, attrition rates from surgical training programmes could be reduced [7]. However, trainees leaving the programme had at least 6 months’ experience of vascular surgery at SHO level, far more than afforded to those currently applying to Improving Surgical Training (IST1) programmes or even most higher specialist training applicants. Junior doctors rotating through vascular surgery should therefore be afforded more opportunity to experience the working patterns of consultants and registrars to gain this realistic insight.

The authors recommend prospective data collection of vascular surgery trainee destinations following exit from the training programme at any stage. We recommend a formal exit interview process, with data being used to feedback nationally into the training programme and interview process, as well as locally to the Deanery involved. To prevent unnecessary resignations at significant personal risk to trainees, we recommend making the IDT system more accessible and flexible. We suggest that ensuring junior doctors have a realistic insight into the working life of vascular surgeons will help to improve the retention of vascular surgical trainees (Table 3).

**Table 3:** Improving vascular surgery trainee retention. Recommendations for improving retention of higher vascular surgery trainees within the training programme, based on suggestions from TPDs’ and trainees’ survey responses

Improving vascular surgery trainee retention	
• Collect data prospectively to quantify resignations • Exit interviews with trainees leaving the specialty to be conducted to identify local and national areas for improvement • Investigate and improve Inter-Deanery Transfer process • Promote an early realistic insight into the lifestyle and working patterns of senior vascular surgeons to junior doctors	

### Limitations

In a small specialty, with a relatively low number of leavers, it is difficult to interrogate the data further whilst maintaining confidentiality and adherence to data protection law. The lack of comparative data from other UK specialties is disappointing. Identification of trainee leavers by existing trainees favoured responses from those trainees still in contact with vascular colleagues, and the lack of centralised recording of trainees who left impacted the low trainee survey response rate. Data collection by a national body such as HEE or the vascular SAC, combined with exit interview data from TPDs could help in future to inform and improve vascular training programme retention.

## Conclusions

This study provides an insight into the nationwide trainee retention rate for a single specialty. Extrapolation of these figures to other surgical specialties would reveal a concerning rate of trainee attrition and we would encourage other specialties to investigate this problem. Work-life balance and system pressures drive trainees to leave a surgical career entirely and although national initiatives will begin to address some of these, we urge TPDs and Deaneries to use exit interviews to address both local and national issues in the form of a feedback loop to inform training and trainee selection. Assessing the outcome of the new IST programmes for selected surgical specialties, including vascular surgery, will be crucial, given these trainees will have made career decisions at a much earlier stage than those already in training.

## Supplementary Information

**Additional file 1.** TPD questionnaire.

**Additional file 2.** Survey to trainees who resigned a vascular surgery NTN between 2013 and 2019.

**Additional file 3.** Ethics Result – England.

**Additional file 4.** Ethics Result - Northern Ireland.

**Additional file 5.** Ethics Result – Scotland.

**Additional file 6.** Ethics Result – Wales.

## Data Availability

The datasets analysed during the current study contain potentially identifying/confidential material so will not be shared.

## Abbreviations

TPDs: Training Programme Directors
NTNs: National Training Numbers
NHS: National Health Service
VSGBI: Vascular Society of Great Britain and Ireland
UK: United Kingdon
US: United States
FOI: Freedom of information
HEE: Health Education England
ENT: Ear, nose and throat
SAC: Specialty advisory committee
ST: Specialty training
OOP: Out of Programme
LTFT: Less than full time
IDT: Inter-deanery transfer
GMC: General Medical Council
SHO: Senior House Officer
IST: Improving surgical training
