# Supplementary material for: Vascular surgery trainee retention in the UK: how many leave and why? A survey of trainee and Training Programme Directors’ perceptions
Source: BMC Med Educ. 2021 Apr 26;21:241. doi: 10.1186/s12909-021-02668-x (PMC8077713; doi:10.1186/s12909-021-02668-x)
Supplement: Supplementary file 1 — Additional file 1. TPD questionnaire. [file 12909_2021_2668_MOESM1_ESM.docx]

**Appendix 1: TPD questionnaire**

We would like to know:

The number of vascular surgery trainees that have left the _______ Deanery in the last 5 years (since the new training number started).

Were these:

-      General surgery trainees that chose vascular surgery?

-      New curriculum trainees?

-      Male or female?

At what stage did they leave?

What reasons for leaving did these people give? We understand the need to preserve confidentiality, so are not asking for individual details, but more main themes, along the lines of the following:

-      Geography

-      Inability to combine a surgical career with family life

-      Pressure, stress or responsibility specific to vascular surgery

-      Switch into a preferred surgical/medical specialty

-      Harassment, bullying or undermining

-      Lacking operative, clinical or decision-making skills to continue

-      Lack of support from supervisors, TPD or other staff

-      Asked to leave the programme

Were any concessions or modifications made to the training programme in order to attempt to keep them in the specialty?

Did any of these trainees have exit interviews? If yes, what useful information did you learn about the drivers to leaving vascular surgery?

Have there been any modifications to any part of the training programme (such as hospitals taken out of the training rotations) on account of any of these trainees?

What do you think could be done within the training programme to help trainees to stay in the specialty?
