# Supplementary material for: Vascular surgery trainee retention in the UK: how many leave and why? A survey of trainee and Training Programme Directors’ perceptions
Source: BMC Med Educ. 2021 Apr 26;21:241. doi: 10.1186/s12909-021-02668-x (PMC8077713; doi:10.1186/s12909-021-02668-x)
Supplement: Supplementary file 2 — Additional file 2. Survey to trainees who resigned a vascular surgery NTN between 2013 and 2019. [file 12909_2021_2668_MOESM2_ESM.docx]

**Appendix 2: Survey to trainees who resigned a vascular surgery NTN between 2013 and 2019**

At what stage did you decide on a career in vascular surgery?

At the time you applied, were you sure it was the career for you?

How much clinical experience of vascular surgery did you have before you applied?

Months of experience at foundation/core level?

Extra courses?

Audits/projects?

SSC/SSM whilst at medical school?

Did you get into vascular surgery training on your first attempt?

Had you had any extra years of clinical practice e.g. locuming/F3?

Did you take any time out of training?

What was it? E.g. maternity leave, OOP, gap year, career break?

At what stage did you decide to leave vascular surgery?

What grade were you when you left?

What were your reasons for leaving?

Did you have an exit interview?

Did you consider taking a period out of programme? If you did, did this help? If you didn’t, was there a reason for this?

Did you consider flexible/LTFT training? If you did, did this help? If you didn’t, was there a reason for this?

Could anything have changed your mind?

Were any solutions to your issues offered?

Would you have continued in vascular surgery if concessions/alterations were made?

What do you think could be done within the training programme to help trainees to stay within the specialty?

Would you ever consider coming back to vascular surgery?

Would you recommend it as a career to others?

What are you doing now?

Are you happy with your new career choice? What does it offer that vascular surgery does not?
